# Spatial Engineering of Gas Diffusion Layers Overcomes Mass Transport Limitations in Fuel Cells

**DOI:** 10.1002/advs.202519772

**Published:** 2026-06-12

**Authors:** Shangwei Zhou, Wenjia Du, Jianuo Chen, Yunsong Wu, Bochen Li, Jieyang Li, Linlin Xu, Winfried Kockelmann, Meng Lin, Marc‐Olivier Coppens, Paul R Shearing, Rhodri Jervis, Thomas S. Miller

**Affiliations:** ^1^ Electrochemical Innovation Lab Department of Chemical Engineering University College London London UK; ^2^ Advanced Propulsion Lab University College London London UK; ^3^ Department of Engineering Science University of Oxford Oxford UK; ^4^ Oxford Martin School University of Oxford Oxford UK; ^5^ The ZERO Institute University of Oxford Oxford UK; ^6^ School of Electrical Engineering Southwest Jiaotong University Chengdu Sichuan China; ^7^ Department of Mechanical and Energy Engineering Southern University of Science and Technology Shenzhen China; ^8^ Centre For Nature‐Inspired Engineering & Department of Chemical Engineering University College London London UK; ^9^ Science and Technology Facilities Council (STFC) Rutherford Appleton Laboratory ISIS Facility Harwell UK

**Keywords:** current density distribution mapping, gas diffusion layer, neutron imaging, PEFC, temperature distribution mapping

## Abstract

Mass transport limitations at high current densities hinder polymer electrolyte fuel cell (PEFC) performance due to inefficient water management and reactant distribution. Gas diffusion layer (GDL) perforation offers a potential solution as an alternative to complex flow‐field modifications. However, understanding of how perforation impacts critical internal states like water distribution, local temperature and electrochemical reaction rates remains limited. This knowledge gap has prevented optimisation of GDL designs, with homogeneous patterns  potentially exacerbating performance gradients. This study uses *operando* neutron imaging combined with synchronous thermal‐electrical mapping to analyse water dynamics and their impact on PEFC performance with both homogeneous and heterogeneous GDLs. The approach enables detailed analysis of interactions between water, heat and electrochemical reactions, providing experimental validation beyond limited field‐of‐view techniques like X‐ray tomography. Results demonstrate that a spatially tailored perforation pattern effectively balances in‐plane saturation gradients and enhances peak power compared to both uniform patterns and non‐perforated GDLs. This work establishes spatial engineering as a design principle for porous transport layers, offering a simpler and cost‐effective solution to mass transport constraints in fuel cells and other electrochemical devices containing porous media.

## Graphical abstract

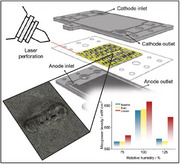

## 1 Introduction

Polymer electrolyte fuel cells (PEFCs) have demonstrated potential as next‐generation power sources for heavy‐duty or long‐distance transportation and stationary applications [1, 2, 3, 4]. While progress has been made on water management, effective and scalable solutions for reactant distribution and water removal remain a critical challenge for the commercial deployment of low‐temperature PEFCs. A key persistent issue is performance loss at high current densities, where product water accumulation can block the pores of the gas diffusion layer (GDL) and catalyst layer (CL) [5], simultaneously hindering the supply of reactants whose consumption rate is high. This confluence of flooded pores and high demand can lead to local reactant starvation, highlighting the need for innovative solutions that are effective at scale. To improve water management and optimise reactant concentration gradients, various flow‐field designs have been proposed to realise uniform reactant distribution, efficient water removal and improved water balance compared to the conventional “land‐channel” configuration of serpentine flow fields. Designs such as porous foam [6, 7, 8], lung‐inspired structures [9] and microchannels [10, 11] enhance mass transport or water removal through better reactant access and capillary pressure mechanisms. However, the fabrication of these complex, often three‐dimensionalchannel architectures typically require specialised and multi‐step manufacturing processes, which incur high costs and low production throughput, hindering large‐scale commercialisation.

Given these issues, a simpler and more cost‐effective approach is needed. One potential solution lies in optimising the GDL as its microstructure directly affects the behaviour and transport of water and reactants before they reach the flow field or catalyst layer [12]. GDL perforation has been proposed to enhance water removal and improve fuel cell performance by reducing mass transport losses of reactants [13, 14, 15]. Importantly, it also requires less effort and time compared to computer numerical control machining for most complex flow field designs. Previous studies using synchrotron X‐ray radiography have captured microscale water dynamics in the immediate vicinity of perforation holes [16, 17]. Despite being conducted in large‐area cells (∼100 cm^2^), the limited field of view of this technique restricts observation to highly localised regions. These investigations revealed that water accumulates in the perforations over time, eventually forming droplets at the channel interface that are ejected into the flow field. Consequently, while this approach provides valuable pore‐scale insights, it cannot assess the impact of perforations on overall water distribution across the entire membrane electrode assembly (MEA). Furthermore, Lin et al. [18] have explored the impact of perforation pattern geometry on performance. That study compared various arrangements, including patterns aligned with the channel flow (“parallel”), across the lands (“vertical”) and at angled orientations (“oblique”). It was found that a dense, quadrilateral‐patterned grid proved most effective, as it created interconnected pathways for liquid water removal, resulting in a reported 50% increase in maximum current density compared to a non‐perforated baseline.

Although perforated GDLs have demonstrated the potential to enhance fuel cell performance by altering key properties such as permeability, diffusivity and thermal conductivity [19], mapping of temperature, water distribution and current density across the entire MEA has thus far been predominantly explored through modelling [18, 20, 21, 22], where simplifications were necessary in this multiscale and multiphase problem. Therefore, the effectiveness of water removal and transport across the whole MEA still requires experimental validation, considering that the liquid water primarily accumulates in the GDLs [23]. Furthermore, while homogeneously perforated GDLs show promise, they still suffer from uneven distribution of reactants and water, and the in‐plane saturation and concentration conditions between the inlet and outlet areas of the cell [24] need to be better balanced. Given that a homogeneous design does not align with the inherently non‐uniform nature of the complex electrochemical reactions inside the fuel cell [25], the MEA architecture must be intelligently and heterogeneously tailored to achieve a more uniform distribution of current density, water content, reactants, partial pressure and mechanical strain during operation [26, 27].

In this study, *operando* neutron imaging is combined with thermal‐electrical mapping to assess how water dynamics affect the performance of PEFCs employing heterogeneously perforated GDLs, as well as those with homogeneous perforations and non‐perforated GDLs. By mapping current density and temperature, the coupled relationship between water, heat and reaction intensity can be more clearly analysed and understood. This approach provides experimental insights into the dynamic interactions within the MEA, contributing to a comprehensive understanding of the role of perforations in optimising PEFC performance. Additionally, it offers valuable insights for improving the performance of electrochemical devices employing porous media [28, 29]. Furthermore, this study demonstrates how heterogeneous designs can effectively enhance performance, paving the way for the optimisation of future fuel cell and electrochemical systems.

## 2 Results and Discussion

### 2.1 PEFC Performance Under Different Relative Humidities

To investigate the effects of GDL perforation on PEFC performance, a custom‐designed MEA and single‐cell configuration were developed, incorporating through‐plane perforations on the cathode‐side GDL. Perforations were introduced using laser micromachining, with controlled dimensions and placement strategies tailored to modulate local mass transport. Details of the MEA fabrication, perforation process and cell assembly are provided in SI‐1 Experimental details.

**FIGURE 1:**
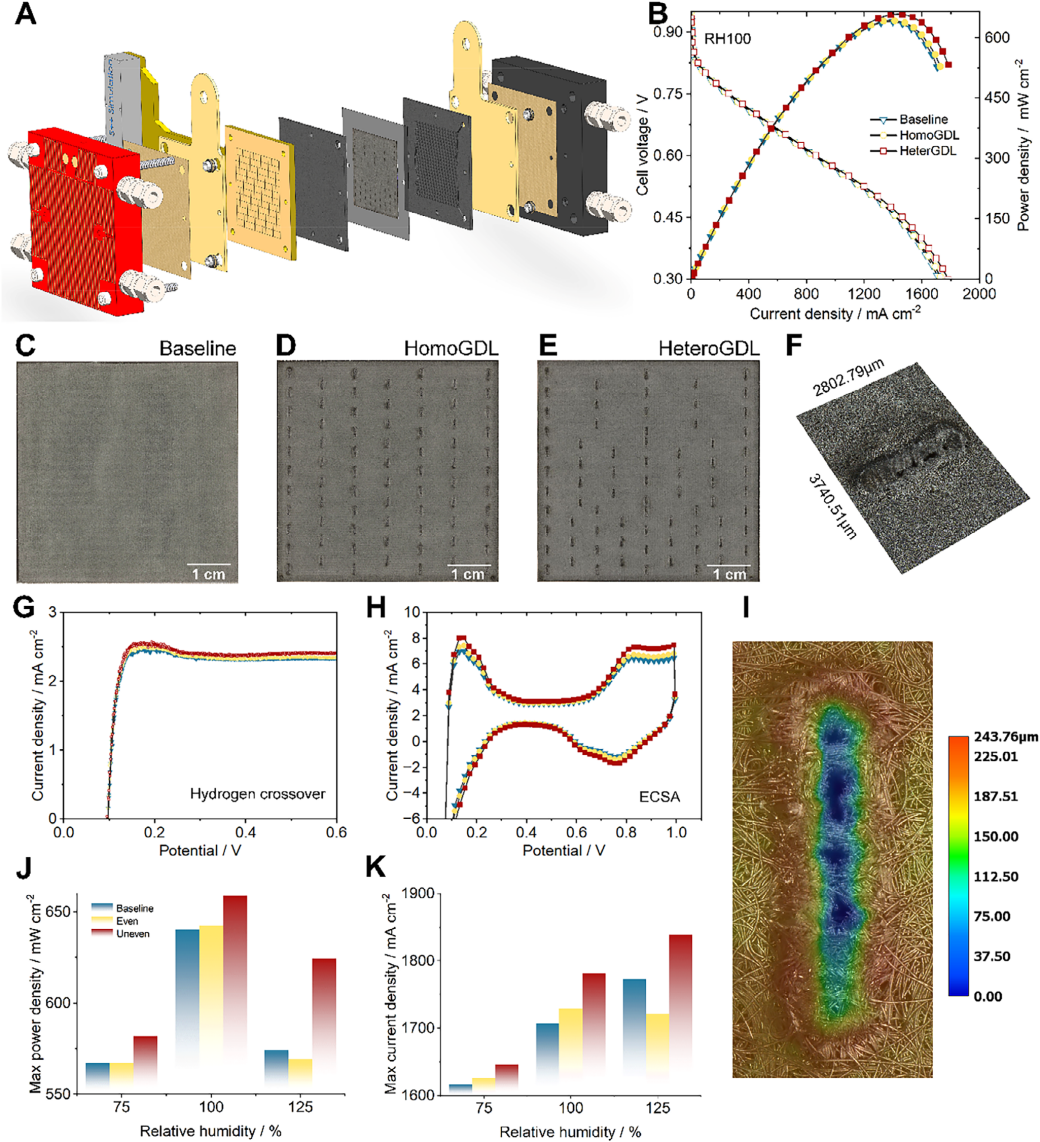
(A) ‘Exploded’ view of the PEFC components before assembly. (B) Cell performance of the baseline, homoGDL and heteroGDL under 100% relative humidity (RH). (C‐E) Top view of the non‐perforated (C), homogeneously perforated (D) and heterogeneously perforated (E) GDLs. (F) Two‐dimensional measurements of the perforated holes using an optical microscope. (G, H) Linear sweep voltammetry (LSV) of the MEAs and cyclic voltammetry (CV) after the break‐in procedure. LSV measurements were conducted with 0.2 L min^−1^ H_2_ supplied to the fuel cell anode and 0.2 L min^−1^ N_2_ supplied to the cathode at 70°C and 100% RH, using a scan rate of 1 mV s^−1^. CV measurements were performed at a scan rate of 20 mV s^−1^. (I) Depth measurement of the perforated holes using a digital microscope. (J) Peak power density under different RHs. (K) Maximum current density (at 0.3 V) under different RHs.

The polarisation curves in Figure 1 compare the baseline (non‐perforated) and homogeneously perforated (homoGDL) GDLs against our novel contribution: a heterogeneously perforated GDL (heteroGDL) designed with a spatially tailored pattern to actively manage in‐plane transport gradients. This design represents a new strategy that moves beyond simple uniform perforation. Since the performance of PEFCs is primarily limited by the oxygen reduction reaction [30], this study focuses on the cathode‐side GDL perforation (as shown in Figure 1A). For the homoGDL (Figure 1D) and heteroGDL (Figure 1E), the size and number of perforations are identical; however, their spatial distribution within the GDL plane has been optimised based on inlet and outlet positions, considering the uneven distribution of humidity and reactant concentrations in the plane [31]. A normal GDL was used to isolate the perforation effect, as the required hydrophobic treatment (∼280°C) would degrade the ionomer in commercial gas diffusion electrode (GDE) and introduce additional variability. In addition, the hydrogen crossover and electrochemically active surface area (ECSA) results shown in Figure 1G,H confirm the consistency of the CL and membrane across all tested samples. The perforation dimensions are shown in Figure 1F, with a length and width of approximately 2.6 mm × 0.6 mm. The 3D profile of the perforations is illustrated in Figure 1I. This configuration achieves a balance between improved performance and mechanical stability, as excessive material removal can compromise the latter. It can be observed that only part of the perforation's central region is fully penetrated by the laser. Additionally, after the experiments, the perforated GDLs were peeled off from the cathode membrane, further demonstrating localised penetration (Figure S1D–F).

At 100% reactant gas RH, the baseline GDL exhibits a maximum power density of 640.28 mW cm^−2^ and a maximum current density (current density at 0.3 V) of 1707.3 mA cm^−2^ (Figure 1B). This performance exceeds that of an MEA using a commercial GDE under similar operating conditions, where the peak power density is 522 mW cm^−2^ [11]. After homogeneous perforation, the maximum power density and maximum current density increase slightly to 642.85 mW cm^−2^ and 1728.5 mA cm^−2^, respectively, even with extra hydrophobic coating. This subtle improvement is attributed to the perforations providing high‐speed pathways for oxygen and liquid water transport within the GDL [18, 22]. This homogeneous design was further optimised by strategically distributing the perforation density to counteract reactant depletion along the channel. Specifically, the density was lowest near the gas inlet, where reactant concentration is high, and water accumulation is less critical. The density was increased toward the outlet, where liquid water tends to accumulate due to higher saturation, and reactant starvation is most likely. This strategy ensures more aggressive water removal in regions where it is most needed, promoting uniform reactant distribution across the entire active area. As a result, the maximum power density and maximum current density reach 659.03 mW cm^−2^ and 1781.5 mA cm^−2^, respectively. Compared to the baseline, the maximum power density is enhanced by 18.75 mW cm^−2^ and the maximum current density by 74.2 mA cm^−2^. Notably, in this study, a performance improvement was achieved solely through the simple heterogeneous perforation of the GDL, which enabled a higher power density and a broader operating current range under the tested conditions. The effectiveness of this approach was demonstrated across a wide range of humidification conditions, showing its strong potential as a scalable solution.

Under different RHs, the performance enhancement due to heterogeneous perforation remains consistent. At 75% reactant RH, the maximum power density increases by about 14.67 mW cm^−2^. Under oversaturated conditions (125% RH), these improvements are about 50.19 mW cm^−2^, respectively. It can be observed that as humidity increases, the magnitude of the peak power density enhancement also significantly increases for heterogeneous perforations. This effect is particularly pronounced under oversaturated conditions, where excessive water accumulation amplifies the importance of effective reactant distribution and improved water management within the cell. Although these observations are based on a single 25 cm^2^ cell, the effects are expected to be even more pronounced in larger cells and stacks in automotive applications, where active water management is more difficult. This also suggests the potential for optimising heterogeneous perforations not only in‐plane but also across the stack. For instance, cells near the end plates may need fewer perforations than those in the middle.

### 2.2 Thermal‐Electro‐Hydro Mapping

**FIGURE 2:**
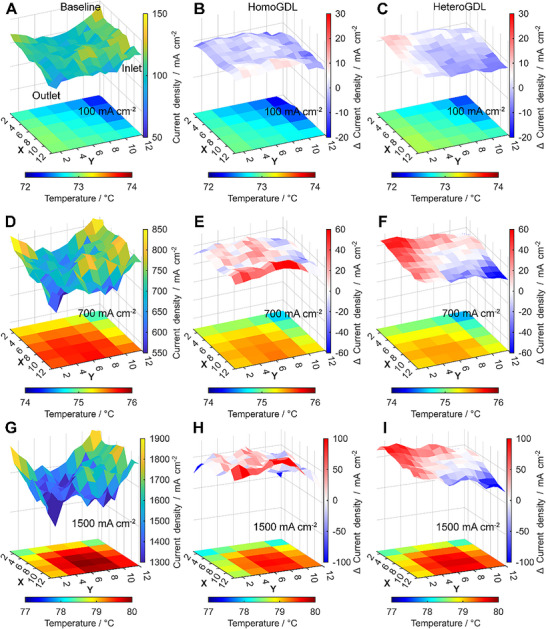
The corresponding current density distribution (vertical axis) and temperature distribution (base plane, rainbow color gradient) for (A) the activation region (j = 100 mA cm^−2^) without perforation. (B,C) The change of current density distribution (compared to no perforation) and temperature distribution corresponds to the activation region under the homogeneous (B) and heterogeneous (C) perforation, respectively. (D) The Ohmic region (j = 700 mA cm^−2^) without perforation., (E,F) The change of current density distribution (compared to no perforation) and temperature distribution corresponds to the Ohmic region under the homogeneous (E) and heterogeneous (F) perforation, respectively., (G) The mass transport controlled (j = 1500 mA cm^−2^) without perforation., (H,I) The change of current density distribution (compared to no perforation) and temperature distribution corresponds to the mass transport controlled under the homogeneous (H) and heterogeneous (I) perforation, respectively. 100% RH, cell temperature of 70°C with hydrogen stoichiometry of 1.5 and air stoichiometry of 3.0.

Although the mechanism of reduced overall liquid water saturation and improved oxygen diffusion in perforated GDLs has been demonstrated through pore‐scale simulations [18], the impact of perforation on the overall reaction distribution within the MEA remains unexplored. The current density and temperature distributions for the baseline, homoGDL and heteroGDL under 100% RH conditions are shown in Figure 2. The current density mapping reflects the in‐plane reaction intensity, with the reactant inlet at (12,12) and the outlet at (12,0).

In the activation region, where the current density is relatively low (100 mA cm^−2^), the baseline (Figure 2A) exhibits a relatively uniform current density distribution, with a standard deviation (STD) of 10.58 mA cm^−2^. This uniform current density distribution corresponds to a uniform temperature distribution [32], with an average temperature of 72.73°C and a standard deviation of 0.27°C. After homogeneous perforation, the change in current density distribution compared to the baseline is relatively subtle (Figure 2B), with a maximum increase of 8.64 mA cm^−^^2^. Similarly, the heterogeneous perforation case (Figure 2C) shows only a limited variation in current density, with a maximum increase of 10.94 mA cm^−^^2^. In both cases, the increase mainly appears in the low‐current‐density regions near the outlet, while the previously high‐current‐density regions show a slight reduction, indicating a more uniform reaction distribution across the MEA. The temperature distributions for the baseline, homoGDL and heteroGDL exhibit similar trends, with relatively higher temperatures observed in the middle and outlet regions. Although overall performance enhancement is not significant at such low current densities, these observations suggest that the perforation design still contributes to improving reaction uniformity and mitigating localised electrochemical activity within the cell [33].

**FIGURE 3:**
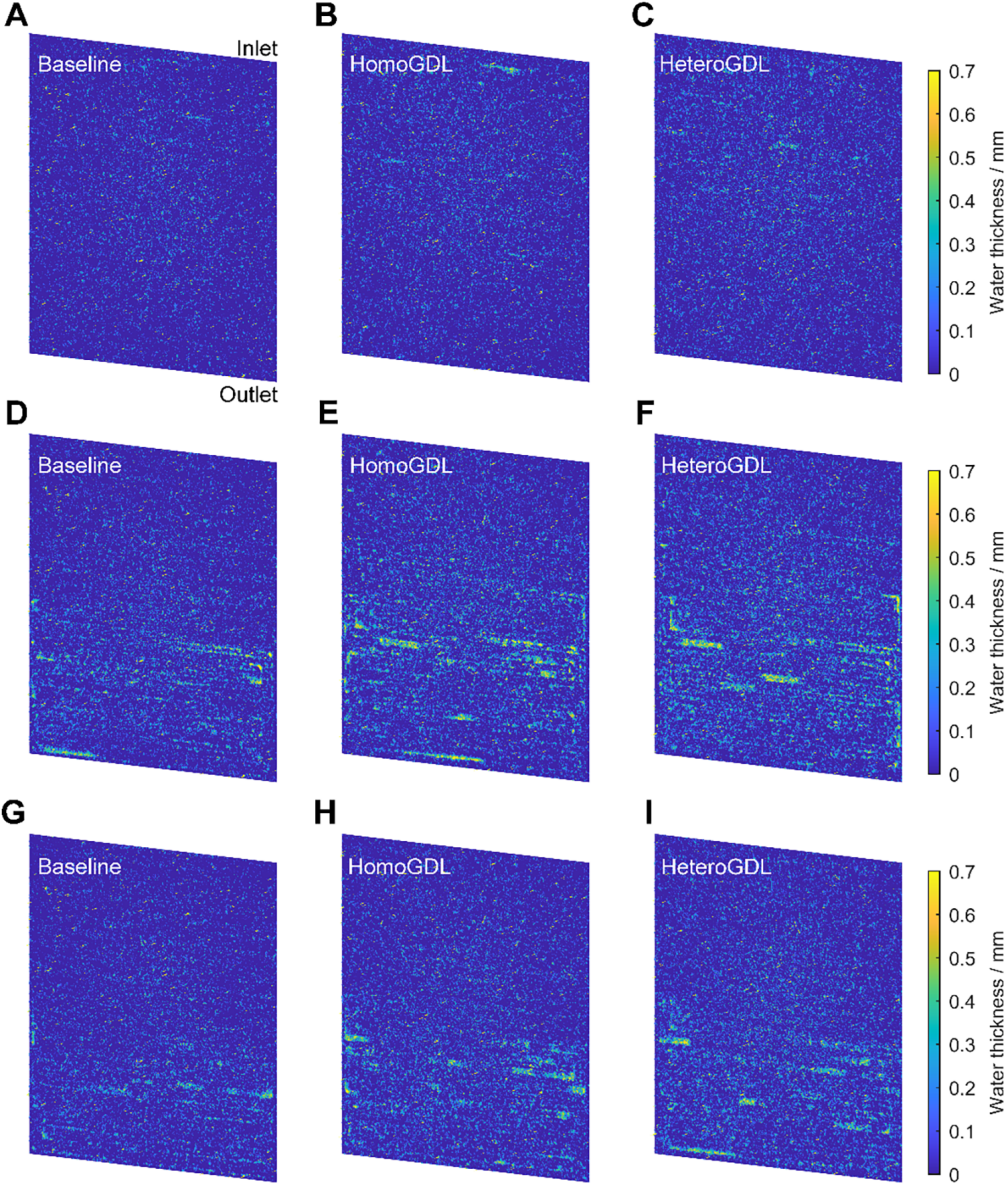
(A–I) Overall water content distribution (through‐plane direction) in the active area corresponding to the activation region under non‐perforation (A), homogeneous (B) and heterogeneous (C) perforation, respectively (100% RH); the Ohmic region under non‐perforation (D), homogeneous (E) and heterogeneous (F) perforation, respectively (100% RH); the mass transport controlled region under non‐perforation (G), homogeneous (H) and heterogeneous (I) perforation, respectively (100% RH). Details of how water thickness was calculated can be seen in the neutron imaging collection and data analysis part of the experimental procedures section.

The corresponding water distribution in the activation region is shown in Figure 3A–C. It can be observed that perforation does not enhance the uniformity of water distribution. The reactant inlet is at the top right, and the outlet is at the bottom right (as shown in Figure S1A). The average water thicknesses for the baseline, homoGDL and heteroGDL are 0.0212, 0.0279, and 0.0327 mm (Table S2), respectively, indicating that more water is retained within the cell rather than being effectively removed for the perforated GDLs. This phenomenon can be attributed to the loss of the GDL's original hydrophobic characteristics during the laser perforation process [16, 34]. The Raman results indicate that laser perforation modified the carbon structure of the GDL, as evidenced by the increased band intensities and the narrowing of the D band (Figure S9). These changes suggest enhanced graphitic ordering and structural reorganisation after laser treatment. Although a post‐perforation hydrophobic treatment was applied, consisting of a brief immersion in a fluorinated ethylene propylene dispersion solution to mitigate excessive Ohmic resistance, it may not have been sufficient to fully restore the GDL's hydrophobicity. Consequently, due to capillary pressure [35], water is drawn into the perforation holes from the surrounding pores, as illustrated in Figure S1C. This could be improved with optimised manufacturing.

At 75% reactant RH, the average water thicknesses for the baseline, homoGDL and heteroGDL are 0.0216 mm (Figure S3A), 0.0253 mm (Figure S3B) and 0.0297 mm (Figure S3C), respectively, also see Table S1. The current density distribution in all perforated cases shows a compensatory effect at the inlet (Figure S2A–C). Given that the average water content within the cell increases after perforation for 75% and 100% inlet RH, this compensation at the inlet can be attributed to improved water retention, particularly considering the low reactant consumption rate in the activation region. At 125% RH (Table S3), the average water content within the cell increases to 0.0338 mm (Figure S6A), 0.0404 mm (Figure S6B) and 0.0440 mm (Figure S6C) for the different GDLs, respectively. It is observed that, even in the baseline case, the current density at the inlet remains lower than that under 75% and 100% inlet RH conditions. Under oversaturated conditions, the influence of heterogeneous perforation on the inlet current density distribution is reduced, decreasing from 10.94 mA cm^−2^ at 75% RH and 7.49 mA cm^−2^ at 100% RH to 6.34 mA cm^−2^ (Figures S2 and S5). This behaviour arises because the inlet region already contains sufficient water to maintain membrane hydration, thereby reducing the benefit of additional humidification. Consequently, under oversaturated conditions, the role of perforation shifts toward enhancing reactant distribution, promoting a more uniform electrochemical reaction across the active area.

In the Ohmic region (700 mA cm^−2^) under 100% inlet RH, the average cell temperature rises to 75.38°C, with a temperature distribution STD of 0.34°C (Figure 2D). Compared to the activation region, reaction non‐uniformity within the MEA increases, and the average water content reaches 0.0360 mm (Figure 3D). Following GDL perforation, a slight performance enhancement is observed compared to the baseline, as indicated by the polarisation curve in Figure 1B. This improvement is attributed to the increasing current density, which accelerates reactant consumption at the triple‐phase boundary. The perforations facilitate more efficient reactant transport to the CL, thereby beginning to mitigate mass transport limitations and enhance overall cell performance. After homogeneous perforation, an increase in current density is observed at the area with low current density (Figure 2E), with a maximum increase of 58.75 mA cm^−2^. Notably, in the localised high‐current‐density region at the corner (0,12), the current density decreases by 124.42 mA cm^−2^ after perforation, indicating that perforation has a ‘peak‐shaving and valley‐filling’ effect on current density distribution. Compared to the baseline, the average water content within the cell increases by 42.8%, reaching 0.0514 mm (Figure 3E). For heteroGDL (Figure 2F), the maximum increase in current density is 52.99 mA cm^−2^, while the localised high‐current‐density region at exactly the cathode inlet (12,12) shows a decrease of 69.12 mA cm^−2^. This demonstrates a stronger peak‐shaving and valley‐filling effect (increased current density over a larger area) on the current density distribution compared to homoGDL. The average cell water content for the non‐uniform perforated GDL is 0.0569 mm (Figure 3F). Despite having the same number of perforations, the higher water content is attributed to the greater concentration of perforations near the outlet [36], enhancing water retention.

At 75% inlet RH (Figure S2D–F), insufficient humidification at the inlet causes an increase in inlet current density to 812.74 mA cm^−2^ compared to 807.55 mA cm^−2^ in Figure 2D at 100% RH. However, after homogeneous perforation, water retention is significantly enhanced at the inlet, compensating for the initial dryness and reducing the local current density by 69.70 mA cm^−2^ (Figure S2E), resulting in a more uniform reaction distribution across the MEA. Notably, this compensatory effect is so effective that the resultant current gain at 75% RH surpasses the gains measured at both 100% (63.94 mA cm^−2^) and 125% RH (55.30 mA cm^−2^), as shown in Figure 2E and Figure S5E. This suggests that, beyond enhancing reactant gas diffusion, perforation also improves local humidification. Under under‐humidified conditions, the improvement in current density distribution results from the combined effects of water retention and reactant facilitation. The enhancement in humidification is evident from the average water content within the cell, measured at 0.0208 mm for the baseline (Figure S3D), 0.0276 mm for the homoGDL (Figure S3E) and 0.0295 mm for the heteroGDL (Figure S3F). Conversely, under over‐humidified conditions of 125% RH, with water distributed throughout the entire cell (Figure S6D–F), the inlet current density compensation effect diminishes for both uniform and non‐uniform perforations, as depicted in Figure S5D–F.

In the mass transport region, corresponding to the current density at maximum power density, a continuous increase in current density distribution non‐uniformity is observed, particularly with a noticeable decline at the outlet (Figure 2G). The average cell temperature rises to 79.18°C, with a temperature STD of 0.57°C. After homogeneous perforation (Figure 2H), the current density at the inlet decreases by 119.81 mA cm^−2^, while for heteroGDL (Figure 2I), this value reaches 131.90 mA cm^−2^. Conversely, the favourable current density compensation that occurred at the outlet location (an increase of 48.96 mA cm^−2^) in the case of heterogeneous perforation, its performance remains superior. The average water content within the cell in the mass transport region is 0.0289, 0.0401, and 0.0459 mm for the baseline (Figure 3G), homoGDL (Figure 3H), and heteroGDL (Figure 3I), respectively. Compared to the Ohmic region, the average water content inside the cell is lower. Although the increase in current density corresponds to higher water production and humidification, the elevated reactant flow rate also enhances water removal. Moreover, the water retention ability of the perforation does not benefit fuel cell performance under well‐humidified conditions.

This conclusion is further supported by the water‐electro‐thermal mapping at 75% humidity, where the average water contents within the cell are 0.0215 mm (Figure S3G), 0.0268 mm (Figure S3H) and 0.0309 mm (Figure S3I) for the baseline, homoGDL, and heteroGDL, respectively. After perforation, the maximum current density increases across the whole MEA, reaching 124.99 mA cm^−2^ for homoGDL (Figure S2H) and 95.62 mA cm^−2^ for heteroGDL (Figure S2I). It is evident that, as the RH increases from 75% to 100%, the compensatory effect of perforation on current density decreases. Under well‐humidified conditions, the performance improvement resulting from perforation is mainly due to enhanced gas transport rather than improved humidification or water management. The liquid water accumulated can improve membrane hydration, but it can also lead to an increase in mass transport resistance, which means mass transport limitations outweigh the benefits of improved membrane hydration.

Additionally, air stoichiometry was varied from 1.5 to 3.0 to investigate the effect of air flow rate. The resulting polarisation curves are shown in Figure S12. The performance differences persist across all air stoichiometries, and the peak‐shaving and valley‐filling effect (Figures S14–S17) is consistently observed under all tested conditions. From the EIS analysis (Figure S13), both the perforated and baseline GDLs exhibit comparable ohmic resistance, indicating that the perforation does not significantly influence membrane hydration or ionic conductivity. Differences between the baseline and perforated become apparent beyond the Ohmic region and are most pronounced in the mass transport regime. In this region, the HeteroGDL demonstrates a reduced impedance in the low‐frequency area compared to the baseline GDL.

A constant current durability test at 1000 mA cm^−2^ for 10 h on both the perforated and baseline GDLs. The results indicate that the perforated GDL does not exhibit additional performance degradation compared to the baseline case (Figure S8) at the earlier degradation. Furthermore, spatially resolved current mapping was employed to investigate local degradation behaviour. For the baseline GDL, as degradation progresses, certain regions become increasingly active, leading to elevated local current densities and a more heterogeneous reaction distribution (Figure S10). In contrast, the perforated GDL does not show this pronounced localisation effect. The current distribution remains comparatively more uniform over time, suggesting that the perforation may help mitigate the development of reaction inhomogeneity during operation (Figure S11).

### 2.3 Localised Analysis

**FIGURE 4:**
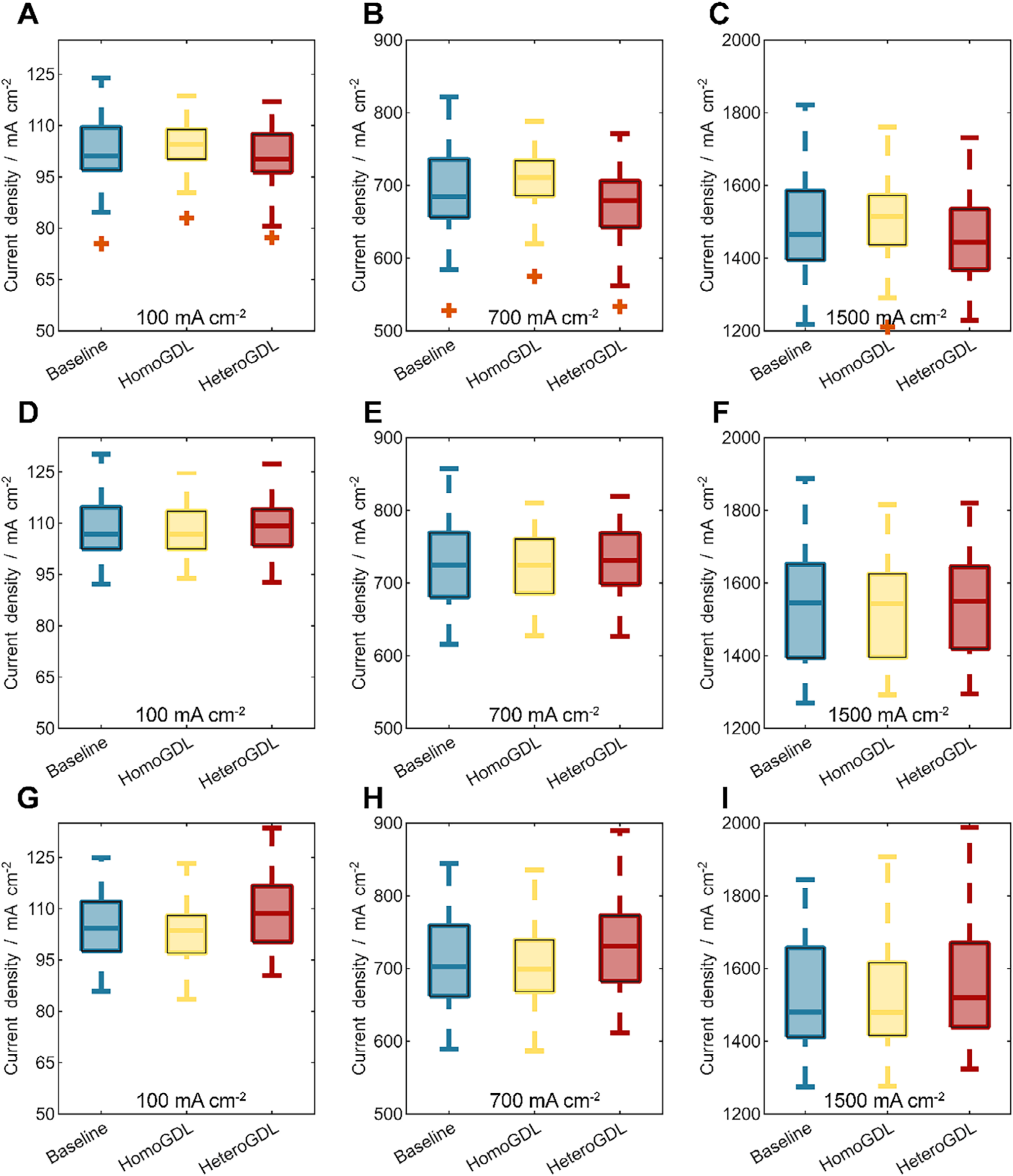
(A–I) Box plot of the current density distribution in the inlet area under the activation region (A), Ohmic region (B) and mass transport‐controlled region (C), respectively (100% RH); the central area under the activation region (D), Ohmic region (E) and mass transport‐controlled region (F), respectively (100% RH); the outlet area under the activation region (G), Ohmic region (H) and mass transport‐controlled region (I), respectively (100% RH).

Based on the spatial distribution of perforations within the MEA plane (Figure 1C–E), the statistical analysis can be categorised into three equally sized areas: the inlet area, the central area, and the outlet area. The inlet area is positioned at the top of the cell, while the outlet area is located at the bottom, where the reactant gases exit. In the inlet area, across all current densities, including the activation region (Figure 4A), the Ohmic region (Figure 4B), and the mass transport region (Figure 4C), statistically changes in current density are observed for both homoGDL and heteroGDL compared to the baseline. Specifically, this enhancement is reflected in the decrease in median current density within the inlet region, indicating less intensified and more evenly distributed electrochemical reaction. In the mass transport region, where performance discrepancies are more pronounced, the heteroGDL demonstrates superior performance relative to homoGDL. This is evidenced by a notable decrease in both the median and maximum current density, and an increase in the minimum current density. Similar trends are consistently observed under both 75% (Figure S4C) and 125% RH conditions (Figure S7C), further confirming the robustness of non‐uniform perforation in enhancing current density across varying operational RH conditions.

In the mass transport region of the central area (Figure 4F), it is observed that after perforation, the minimum current density increases. Conversely, in the outlet area (Figure 4I, Figures S4I and S7I), the current density distribution exhibits an overall increasing, slight trend across various humidity conditions, with both uniform and non‐uniform perforations. Notably, in the mass transport region, perforation leads to a high maximum and minimum current density, significantly more homogeneous compared to the non‐perforated baseline.

## 3 Conclusions

This study comprehensively analysed the impact of heterogeneously perforated GDLs on PEFC performance through *operando* neutron imaging and thermal‐electrical mapping. By evaluating water, temperature and current density distributions across the entire cell, the mechanism by which perforation patterns influence oxygen transport and electrochemical performance under different RH conditions was revealed. At 100% RH, heterogeneous perforation improved water retention and reactant distribution, increasing peak power density by 18.75 mW cm^−2^ compared to non‐perforated baselines, while homogeneous perforation achieved similar performance results from extra coating. Similarly, at 75% RH, heterogeneous GDLs boosted peak power density by 14.67 mW cm^−2^, with the same catalyst loading, achived solely through GDL perforation. Under oversaturated conditions (125% RH), where liquid water accumulation dominates mass transport losses, heterogeneous perforation more effectively mitigated local reactant depletion, increasing maximum power density by 50.19 mW cm^−2^ compared to a 5.23 mW cm^−2^ decrease with homogeneous perforation.

These findings demonstrate the critical role of spatially tailored perforation in optimising oxygen transport and reactant distribution. Under moderate and dry conditions, it balances in‐plane reactant supply and improves local water management, while under oversaturated conditions, it alleviates reactant starvation through superior water removal and gas accessibility. Current density mapping confirmed a ‘peak shaving and valley filling’ effect, smoothing localised high‐current‐density regions and enhancing durability by mitigating reactant depletion and associated degradation. These effects are expected to be even more pronounced in larger‐area cells and stacks, highlighting the potential for optimising heterogeneous perforation designs both in‐plane and through‐plane. Furthermore, this strategy extends beyond PEFCs to electrolysers and other electrochemical systems employing porous media. By enabling more rational design of porous transport layers, this approach offers a cost‐effective, scalable solution for improving water management and reactant utilisation, ultimately advancing the performance and durability of energy conversion technologies.

## Author Contributions

**Shangwei Zhou**: Conceptualization, Methodology, Formal analysis, Investigation, Data curation, Writing – original draft, Writing – review & editing, Visualization. **Wenjia Du**: Investigation, Writing – review & editing. **Jianuo Chen**: Investigation, Writing – review & editing. **Yunsong Wu**: Investigation, Writing – review & editing. **Bochen Li**: Investigation, Writing – review & editing. **Jieyang Li**: Investigation, Writing – review & editing. **Linlin Xu**: Conceptualization, Writing – review & editing. **Winfried Kockelmann**: Investigation, Writing – original draft, Writing – review & editing. **Meng Lin**: Writing – review & editing, Supervision. **Marc‐Olivier Coppens**: Writing – review & editing, Supervision. **Paul R Shearing**: Writing – review & editing, Supervision. **Rhodri Jervis**: Resources, Writing – review & editing, Supervision. **Thomas S. Miller**: Conceptualization, Resources, Writing – original draft, Writing – review & editing, Supervision, Project administration, Funding acquisition.

## Conflicts of Interest

The authors declare no conflicts of interest.

## Supporting information




**Supporting File**: advs76037‐sup‐0001‐SuppMat.docx.

## Data Availability

The data that support the findings of this study are available from the corresponding author upon reasonable request.
