# Supplementary material for: Spatial Engineering of Gas Diffusion Layers Overcomes Mass Transport Limitations in Fuel Cells
Source: Adv Sci (Weinh). 2026 Jun 12;13(50):e19772. doi: 10.1002/advs.202519772 (PMC13336354; doi:10.1002/advs.202519772)
Supplement: Supplementary file 1 [file ADVS-13-e19772-s001.docx]

**Supplemental information**

**Spatial Engineering of Gas Diffusion Layers Overcomes Mass Transport Limitations in Fuel Cells**

Shangwei Zhou ^a, b,^ *, Wenjia Du ^c, d, e^, Jianuo Chen ^a^, Yunsong Wu ^f^, Bochen Li ^a^, Jieyang Li ^a, g^, Linlin Xu ^a, h^, Winfried Kockelmann ^i^, Meng Lin ^g^, Marc-Olivier Coppens ^h^, Paul R Shearing ^c, d, e^, Rhodri Jervis ^a, b^, and Thomas S. Miller ^a, b,^ *

^a^ Electrochemical Innovation Lab, Department of Chemical Engineering, University College London, London WC1E 7JE, UK

^b^ Advanced Propulsion Lab, University College London, London, E20 2AE, UK

^c^ Department of Engineering Science, University of Oxford, Parks Road, Oxford OX1 3PJ, UK

^d^ Oxford Martin School, University of Oxford, Oxford OX1 3BD, UK

^e^ The ZERO Institute, University of Oxford, Oxford OX2 0ES, UK

^f^ School of Electrical Engineering, Southwest Jiaotong University, Chengdu, Sichuan Province 611756, China

^g^ Department of Mechanical and Energy Engineering, Southern University of Science and Technology, Shenzhen 518055, China

^h^ Centre for Nature-Inspired Engineering & Department of Chemical Engineering, University College London, London WC1E 7JE, UK

^i^ Science and Technology Facilities Council (STFC), Rutherford Appleton Laboratory, ISIS Facility, Harwell OX11 0QX, UK

* Corresponding author.

E-mail address: shangwei.zhou.20@ucl.ac.uk, t.miller@ucl.ac.uk

*Keywords*: PEFC; Neutron Imaging; Gas Diffusion Layer; Current Density Distribution Mapping; Temperature Distribution Mapping

**SI-1 Experimental details**

**Ink Preparation and Spray Coating**

The catalyst ink was prepared using 40% platinum supported on high surface area Ketjenblack EC-300J (Fuel Cell Store). The ionomer-to-carbon (I/C) ratio was maintained at 0.8 [1] to balance the ionic conductivity and Ohmic resistance of the catalyst layer. A mixed solvent consisting of deionised water and IPA in a weight ratio of 6:4 was used to facilitate uniform dispersion, with the solid content set at 5 mg/ml to prevent nozzle blockage during the spray coating process. The ionomer dispersion used in this work was Aquivion® D72-25BS. The catalyst ink was initially homogenised using a vortex mixer (RX3, Velp Scientifica) for 30 seconds, followed by ultrasonication (VWR^®^, USC900TH) in an ice bath for 30 minutes to ensure stable and thorough dispersion.

For the preparation of the half-cell catalyst-coated membrane (half-CCM), a Gore Select membrane (GORE M765.08, USA) was placed flat on the heated plate of a Sono-Tek Exacta-coat equipped with dual nozzles. The two spray nozzles were arranged at an angle of 25° to ensure uniform coating distribution. The temperature of the heated plate was set to 80 °C to facilitate rapid solvent evaporation during the spraying process. The catalyst ink was sprayed at a flow rate of 0.1 L/min for each nozzle, with the shaping air pressure maintained at 1 bar. The membrane was secured under an acrylic gasket featuring a 5 cm × 5 cm cutout to define the coating area. To determine the Pt loading, the membrane was weighed before and after the spray coating process with the high-capacity microbalance (Sartorius MCA66S-3S00-D), with the difference in mass corresponding to the deposited catalyst.

**Laser Perforation and MEA Hot Compression**

The 250 μm-thick Freudenberg H23C6 carbon paper with an MPL was cut into 5 cm × 5 cm squares and placed inside a Hobart laser cutter. The laser cutting parameters were set to 90% power and 18% speed, with the Z-axis positioned at a height of 1.4 mm. The perforation template was designed using Rhinoceros^®^ and executed in three cutting passes. After cutting, dry air was used to remove any surface residues, ensuring a clean and debris-free surface.

Subsequently, the perforated GDLs were immersed in the 3/5 wt% FEPD solution for 1 minute, then dried in a vacuum oven at 60 °C for 1 hour to remove water and other solvents. To eliminate the surfactant, the GDLs were heated in a muffle oven with the temperature gradually increased to 260 °C over 50 minutes, followed by a 10-minute hold at 260 °C. Subsequently, to sinter the polymer, the temperature was raised to 280 °C over 20 minutes and maintained at 280 °C for an additional 20 minutes [2].

The half-CCM was assembled by sandwiching it between a GDL serving as the cathode and a commercial gas diffusion electrode (GDE) as the anode. The commercial GDE (Hyplat, South Africa) featured a Pt loading of 0.4 mg cm^-2^ and was based on Freudenberg H23C9 carbon paper with a 15 μm microporous layer (MPL) subjected to hydrophobic treatment. The membrane electrode assembly (MEA) was then positioned between steel plates and hot-pressed using a Carver hot press (USA) at 150 °C for 3 minutes under a compression force of 1000 psi. To prevent mechanical damage and avoid GDE displacement on both sides, Kapton film sheets were placed between the MEA and the steel plates.

**Fuel cell configuration and experimental setup**

A custom-designed single PEFC with a 25 cm² active area was designed for through-plane neutron imaging, with the MEA positioned perpendicular to the neutron beam. To minimise neutron absorption, 20 mm-thick aluminium endplates were used, with a PTFE sheet placed between the anodised endplate and the gold-coated current collector for electrical insulation. The graphite flow-field plates, 2 mm in thickness, featured a two-channel serpentine design with channel and rib dimensions of approximately 1 mm in width and 1 mm in depth. Kapton flexible heaters were affixed to both sides of the endplates to ensure uniform heating, preventing local hotspots and vapour condensation. PTFE gaskets maintained gas tightness while also allowing for proper MEA compression. Four bolts at the corners of the endplates provided uniform compression, gradually torqued to 2 N·m. Gases were introduced perpendicular to the neutron beam, enabling the fuel cell’s position near the neutron camera box for enhanced neutron imaging quality.

The fuel cells were operated at 70°C using a commercial Scribner 850e test bench, ensuring precise temperature and humidification regulation. Hydrogen and air were supplied at stoichiometric ratios of 1.5 and 3, respectively, with no applied back pressure. High-purity gases were utilised, including nitrogen (99.9%), air (99.9%), and hydrogen (99.995%), to maintain consistent operating conditions. In addition, supplementary experiments were conducted under DoE-recommended operating conditions to further evaluate the performance of the modified GDLs. These tests were performed at 80 °C and 100% RH, with hydrogen stoichiometry fixed at 1.2 and air stoichiometry varied from 1.5 to 3.0.

**Raman spectroscopy**

Raman spectroscopy was performed to investigate the geometric effects of perforation or laser-induced chemical modification of the carbon surface. The measurements were carried out using a 532 nm excitation laser with a power of 10 mW, a 50 μm slit aperture, and a 900 lines/mm grating, providing an estimated spectral resolution of 5.5-8.3 cm^-1^ over a range of 18-3566 cm^-1^.

**Neutron imaging and data analysis**

Neutron imaging was performed at IMAT (**Fig. S1A**), ISIS Neutron and Muon Source, UK. The IMAT setup featured a collimator with a diameter (D) of 30 mm, positioned 10.45 m upstream of the neutron detector, resulting in a quasi-parallel beam with a collimation ratio of L/D = 348. A Tritec ZnS/LiF:Cu scintillator screen with a thickness of 100 μm was used to convert neutrons with wavelengths ranging from 0.7 to 6.7 A into visible light. The scintillator screen, 120 × 120 mm², was mounted inside a black-anodised aluminium box facing the incident neutron beam. A mirror placed at a 45° angle relative to the scintillator reflected the emitted light out of the neutron beam path. This light was then directed through a Nikon F/2.0 lens onto an ANDOR IKON-L CCD digital camera with a resolution of 2,048 × 2,048 pixels, providing an imaging field of view of approximately 98 × 98 mm² and a pixel size of 48 μm. The spatial resolution was limited to 115 μm due to the L/D collimator ratio and a cell-to-scintillator distance of 40 mm. An exposure time of 10 seconds was selected to achieve an adequate signal-to-noise ratio.

The water thickness from the neutron images was determined using the Beer-Lambert law [3]:

$\delta\left( x,y \right)=\frac{-ln(I(x,y)/I_{0}(x,y))}{\mu}$	(1)

where $\delta$ represents the water thickness at a given pixel position $\left( x,y \right)$ in cm, and $\mu$ = 5.3 cm⁻¹ is the neutron attenuation coefficient in liquid water. $I_{0}$ denotes the intensity of the dry image captured prior to gas supply (**Fig. S1B**), while $I$ represents the intensity of the wet image acquired during operation. Image processing and analysis were conducted using the ImageJ. The total water content for each frame was calculated as follows:

$H={\sum_{i=1}^{N_{x}} \sum_{j=1}^{N_{y}} \delta\left( x_{i},y_{i} \right)}/{N_{x}N_{y}}$	(2)

where H is the average water content in mm and $N_{x}$ and $N_{y}$ are the numbers of pixels in the x direction and y direction, respectively.

**Digital microscope and thermal-electro mapping**

To analyse and contrast the fuel cells on a microscopic scale, the Keyence VHX-7100 digital microscope was employed. The VHX-E20 lens provides x20 to x100 magnification. The microscope's integrated software enables two-dimensional measurements, including distances and radii, at any magnification, while also generating depth-focused images [4].

The current and temperature distribution across the 25 cm² PEFC active area was measured using a PCB board (S++ Simulation Services) featuring a 12 × 12 array of shunt resistors and a 6 × 6 grid of temperature sensors. Data acquisition occurred at 100 ms intervals. To minimise measurement artefacts caused by uneven electrical contact, a carbon paper layer was inserted between the PCB board and the cathode current collector. The sensor assembly was positioned at the interface between the cathode flow plate and the current collector.

**References**

[1] Zhao Q, Morawietz T, Gazdzicki P, Friedrich KA. Strategy to tune properties of PEM fuel cell electrodes with low Pt loading based on inkjet printing parameters. Journal of Power Sources. 2025;625:235624.

[2] Hasanpour S, Ahadi M, Bahrami M, Djilali N, Akbari M. Woven gas diffusion layers for polymer electrolyte membrane fuel cells: Liquid water transport and conductivity trade-offs. Journal of Power Sources. 2018;403:192-8.

[3] Zhou S, Wu Y, Xu L, Kockelmann W, Rasha L, Du W, et al. Water content estimation in polymer electrolyte fuel cells using synchronous electrochemical impedance spectroscopy and neutron imaging. Cell Reports Physical Science. 2024;5.

[4] Von Tettau P, Sterlepper S, Mauermann P, Wick M, Tinz S, Jesser M, et al. Laboratory assessments applied to mass-produced automotive fuel cells. International Journal of Hydrogen Energy. 2024;52:1127-36.

**SI-2 Supplementary Figures and Tables**

**Fig. S1*.*** (**A**) Neutron radiography set-up at IMAT to generate in-*operando* neutron imaging in the through-plane orientation. (**B**) Neutron radiograph of a dry and non-operational fuel cell. (**C**) The accumulated water inside the perforated holes after the test. (**D**–**F**) Microporous layer views after the test, where perforated GDLs were peeled off from the cathode membrane: baseline (**D**), homoGDL (**E**), and heteroGDL (**F**). (**G**–**I**) Catalyst layer views after the test: baseline (**G**), homoGDL (**H**), and heteroGDL (**I**).

**Fig. S2.** The corresponding current density distribution (vertical axis) and temperature distribution (base plane, rainbow colour gradient) for (**A**) the activation region (j = 100 mA cm^−2^) without perforation. (**B**–**C**) The change of current density distribution (compared to no perforation) and temperature distribution corresponds to the activation region under the homogeneous (**B**) and heterogeneous (**C**) perforation, respectively. (**D**) The Ohmic region (j = 700 mA cm^−2^) without perforation. (**E**–**F**) The change of current density distribution (compared to no perforation) and temperature distribution corresponds to the Ohmic region under the homogeneous (**E**) and heterogeneous (**F**) perforation, respectively. (**G**) The mass transport controlled (j = 1500 mA cm^−2^) without perforation. (**H**–**I**) The change of current density distribution (compared to no perforation) and temperature distribution corresponds to the mass transport controlled under the homogeneous (**H**) and heterogeneous (**I**) perforation, respectively. 75% RH, cell temperature of 70 °C with hydrogen stoichiometry of 1.5 and air stoichiometry of 3.0.

**Fig. S3**. (**A**-**I**) Overall water content distribution (through-plane direction) in the active area corresponding to the activation region under non-perforation (**A**), homogeneous (**B**) and heterogeneous (**C**) perforation, respectively (75% RH); the Ohmic region under non-perforation (**D**), homogeneous (**E**) and heterogeneous (**F**) perforation, respectively (75% RH); the mass transport controlled under non-perforation (**G**), homogeneous (**H**) and heterogeneous (**I**) perforation, respectively (75% RH).

**Fig. S4.** (**A**-**I**) Box plot of the current density distribution in the inlet area under the activation region (**A**), Ohmic region (**B**), and mass transport-controlled region (**C**), respectively (75% RH); the central area under the activation region (**D**), Ohmic region (**E**), and mass transport-controlled region (**F**), respectively (75% RH); the outlet area under the activation region (**G**), Ohmic region (**H**), and mass transport-controlled region (**I**), respectively (75% RH).

**Fig. S5.** The corresponding current density distribution (vertical axis) and temperature distribution (base plane, rainbow colour gradient) for (**A**) the activation region (j = 100 mA cm^−2^) without perforation. (**B**–**C**) The change of current density distribution (compared to no perforation) and temperature distribution corresponds to the activation region under the homogeneous (**B**) and heterogeneous (**C**) perforation, respectively. (**D**) The Ohmic region (j = 700 mA cm^−2^) without perforation. (**E**–**F**) The change of current density distribution (compared to no perforation) and temperature distribution corresponds to the Ohmic region under the homogeneous (**E**) and heterogeneous (**F**) perforation, respectively. (**G**) The mass transport controlled (j = 1500 mA cm^−2^) without perforation. (**H**–**I**) The change of current density distribution (compared to no perforation) and temperature distribution corresponds to the mass transport controlled under the homogeneous (**H**) and heterogeneous (**I**) perforation, respectively. 125% RH, cell temperature of 70 °C with hydrogen stoichiometry of 1.5 and air stoichiometry of 3.0.

**Fig. S6**. (**A**-**I**) Overall water content distribution (through-plane direction) in the active area corresponding to the activation region under non-perforation (**A**), homogeneous (**B**) and heterogeneous (**C**) perforation, respectively (125% RH). the Ohmic region under non-perforation (**D**), homogeneous (**E**) and heterogeneous (**F**) perforation, respectively (125% RH). the mass transport controlled under non-perforation (**G**), homogeneous (**H**) and heterogeneous (**I**) perforation, respectively (125% RH).

**Fig. S7.** (**A**-**I**) Box plot of the current density distribution in the inlet area under the activation region (**A**), Ohmic region (**B**), and mass transport-controlled region (**C**), respectively (125% RH); the central area under the activation region (**D**), Ohmic region (**E**), and mass transport-controlled region (**F**), respectively (125% RH); the outlet area under the activation region (**G**), Ohmic region (**H**), and mass transport-controlled region (**I**), respectively (125% RH).

**Fig. S8.** (**A**–**B**) Cell voltage evolution (**A**) and corresponding voltage change relative to the initial value (**B**) during a 10 h constant-current durability test at 1000 mA cm^-2^ for the baseline, homoGDL and heteroGDL.

**Fig. S9.** Raman Analysis of Graphitic Structural Changes in Laser-Perforated GDL.

**Fig. S10.** (**A**-**I**) The corresponding current density distribution (vertical axis) and temperature distribution (base plane, rainbow colour gradient) during the durability test for the Baseline at (**A**) 0 h (reference), (**B**) 1.25 h, (**C**) 2.5 h, (**D**) 3.75 h, (**E**) 5 h, (**F**) 6.25 h, (**G**) 7.5 h, (**H**) 8.75 h, and (**I**) 10 h under 1000 mA cm^-1^ constant-current operation. Tests were conducted at 100% RH, cell temperature of 80 °C, hydrogen stoichiometry of 1.2, and air stoichiometry of 2.0.

**Fig. S11.** (**A**-**I**) The corresponding current density distribution (vertical axis) and temperature distribution (base plane, rainbow colour gradient) during the durability test for the HeteroGDL at (**A**) 0 h (reference), (**B**) 1.25 h, (**C**) 2.5 h, (**D**) 3.75 h, (**E**) 5 h, (**F**) 6.25 h, (**G**) 7.5 h, (**H**) 8.75 h, and (**I**) 10 h under 1000 mA cm^-1^ constant-current operation. Tests were conducted at 100% RH, cell temperature of 80 °C, hydrogen stoichiometry of 1.2, and air stoichiometry of 2.0.

**Fig. S12.** (**A**-**I**) Cell performance of baseline, homoGDL, and heteroGDL at 100% RH and 80 °C, with hydrogen stoich 1.2 and air stoich of 1.5 (**A**), 2.0 (**B**), 2.5 (**C**), and 3.0 (**D**).

**Fig. S13.** (**A**–**C**) Impedance comparison of the baseline, homoGDL and heteroGDL in the mass transport-controlled region (**A**), ohmic region (**B**) and activation region (**C**) at an air stoichiometry of 3.0. (**D**–**F**) Impedance comparison of the baseline, homoGDL and heteroGDL in the mass transport-controlled region (**D**), ohmic region (**E**), and activation region (**F**) at an air stoichiometry of 2.5.

**Fig. S14.** The corresponding current density distribution (vertical axis) and temperature distribution (base plane, rainbow colour gradient) for (**A**) the activation region (j = 100 mA cm^−2^) without perforation. (**B**–**C**) The change of current density distribution (compared to no perforation) and temperature distribution corresponds to the activation region under the homogeneous (**B**) and heterogeneous (**C**) perforation, respectively. (**D**) The Ohmic region (j = 700 mA cm^−2^) without perforation. (**E**–**F**) The change of current density distribution (compared to no perforation) and temperature distribution corresponds to the Ohmic region under the homogeneous (**E**) and heterogeneous (**F**) perforation, respectively. 100% RH, cell temperature of 80 °C with hydrogen stoichiometry of 1.2 and air stoichiometry of 1.5.

**Fig. S15.** The corresponding current density distribution (vertical axis) and temperature distribution (base plane, rainbow colour gradient) for (**A**) the activation region (j = 100 mA cm^−2^) without perforation. (**B**–**C**) The change of current density distribution (compared to no perforation) and temperature distribution corresponds to the activation region under the homogeneous (**B**) and heterogeneous (**C**) perforation, respectively. (**D**) The Ohmic region (j = 700 mA cm^−2^) without perforation, (**E**–**F**) The change of current density distribution (compared to no perforation) and temperature distribution corresponds to the Ohmic region under the homogeneous (**E**) and heterogeneous (**F**) perforation, respectively. 100% RH, cell temperature of 80 °C with hydrogen stoichiometry of 1.2 and air stoichiometry of 2.0.

**Fig. S16.** The corresponding current density distribution (vertical axis) and temperature distribution (base plane, rainbow colour gradient) for (**A**) the activation region (j = 100 mA cm^−2^) without perforation. (**B**–**C**) The change of current density distribution (compared to no perforation) and temperature distribution corresponds to the activation region under the homogeneous (**B**) and heterogeneous (**C**) perforation, respectively. (**D**) The Ohmic region (j = 700 mA cm^−2^) without perforation, (**E**–**F**) The change of current density distribution (compared to no perforation) and temperature distribution corresponds to the Ohmic region under the homogeneous (**E**) and heterogeneous (**F**) perforation, respectively. (**G**) The mass transport controlled (j = 1500 mA cm^−2^) without perforation. (**H**–**I**) The change of current density distribution (compared to no perforation) and temperature distribution corresponds to the mass transport controlled under the homogeneous (**H**) and heterogeneous (**I**) perforation, respectively. 100% RH, cell temperature of 80 °C with hydrogen stoichiometry of 1.2 and air stoichiometry of 2.5.

**Fig. S17.** The corresponding current density distribution (vertical axis) and temperature distribution (base plane, rainbow colour gradient) for (**A**) the activation region (j = 100 mA cm^−2^) without perforation. (**B**–**C**) The change of current density distribution (compared to no perforation) and temperature distribution corresponds to the activation region under the homogeneous (**B**) and heterogeneous (**C**) perforation, respectively. (**D**) The Ohmic region (j = 700 mA cm^−2^) without perforation, (**E**–**F**) The change of current density distribution (compared to no perforation) and temperature distribution corresponds to the Ohmic region under the homogeneous (**E**) and heterogeneous (**F**) perforation, respectively. (**G**) The mass transport controlled (j = 1500 mA cm^−2^) without perforation, (**H**–**I**) The change of current density distribution (compared to no perforation) and temperature distribution corresponds to the mass transport controlled under the homogeneous (**H**) and heterogeneous (**I**) perforation, respectively. 100% RH, cell temperature of 80 °C with hydrogen stoichiometry of 1.2 and air stoichiometry of 3.0.

**Table S1**. Average through-plane water content distribution in the active area at 75% RH, cell temperature of 70 °C, hydrogen stoichiometry of 1.5, and air stoichiometry of 3.0.

|  | Baseline | HomoGDL | HeteroGDL |
| --- | --- | --- | --- |
| Activation region | 0.0216 mm | 0.0253 mm | 0.0297 mm |
| Ohmic region | 0.0208 mm | 0.0276 mm | 0.0295 mm |
| Mass transport-controlled region | 0.0215 mm | 0.0268 mm | 0.0309 mm |

**Table S2**. Average through-plane water content distribution in the active area at 100% RH, cell temperature of 70 °C, hydrogen stoichiometry of 1.5, and air stoichiometry of 3.0.

|  | Baseline | HomoGDL | HeteroGDL |
| --- | --- | --- | --- |
| Activation region | 0.0212 mm | 0.0279 mm | 0.0327 mm |
| Ohmic region | 0.0360 mm | 0.0514 mm | 0.0569 mm |
| Mass transport-controlled region | 0.0289 mm | 0.0401 mm | 0.0459 mm |

**Table S3**. Average through-plane water content distribution in the active area at 125% RH, cell temperature of 70 °C, hydrogen stoichiometry of 1.5, and air stoichiometry of 3.0.

|  | Baseline | HomoGDL | HeteroGDL |
| --- | --- | --- | --- |
| Activation region | 0.0338 mm | 0.0404 mm | 0.0440 mm |
| Ohmic region | 0.0360 mm | 0.0470 mm | 0.0573 mm |
| Mass transport-controlled region | 0.0329 mm | 0.0464 mm | 0.0498 mm |
